# Naïve Regulatory T Cell Subset Is Altered in X-Linked Agammaglobulinemia

**DOI:** 10.3389/fimmu.2021.697307

**Published:** 2021-08-19

**Authors:** Pavel V. Shelyakin, Ksenia R. Lupyr, Evgeny S. Egorov, Ilya A. Kofiadi, Dmitriy B. Staroverov, Sofya A. Kasatskaya, Valeriia V. Kriukova, Irina A. Shagina, Ekaterina M. Merzlyak, Tatiana O. Nakonechnaya, Elena A. Latysheva, Irina A. Manto, Musa R. Khaitov, Sergey A. Lukyanov, Dmitriy M. Chudakov, Olga V. Britanova

**Affiliations:** ^1^Shemyakin-Ovchinnikov Institute of Bioorganic Chemistry, Russian Academy of Sciences, Moscow, Russia; ^2^Center of Life Sciences, Skolkovo Institute of Science and Technology, Moscow, Russia; ^3^FSBI “NRC Institute of Immunology” FMBA of Russia, Moscow, Russia; ^4^Institute of Translational Medicine, Pirogov Russian National Research Medical University, Moscow, Russia

**Keywords:** X-linked agammaglobulinemia (XLA), TCR repertoire, T cell gene expression, naïve regulatory T cells, CDR3β features

## Abstract

The interplay between T- and B-cell compartments during naïve, effector and memory T cell maturation is critical for a balanced immune response. Primary B-cell immunodeficiency arising from X-linked agammaglobulinemia (XLA) offers a model to explore B cell impact on T cell subsets, starting from the thymic selection. Here we investigated characteristics of naïve and effector T cell subsets in XLA patients, revealing prominent alterations in the corresponding T-cell receptor (TCR) repertoires. We observed immunosenescence in terms of decreased diversity of naïve CD4^+^ and CD8^+^ TCR repertoires in XLA donors. The most substantial alterations were found within naïve CD4^+^ subsets, and we have investigated these in greater detail. In particular, increased clonality and convergence, along with shorter CDR3 regions, suggested narrower focused antigen-specific maturation of thymus-derived naïve T_reg_ (CD4^+^CD45RA^+^CD27^+^CD25^+^) in the absence of B cells - normally presenting diverse self and commensal antigens. The naïve T_reg_ proportion among naïve CD4 T cells was decreased in XLA patients, supporting the concept of impaired thymic naïve T_reg_ selection. Furthermore, the naïve T_reg_ subset showed prominent differences at the transcriptome level, including increased expression of genes specific for antigen-presenting and myeloid cells. Altogether, our findings suggest active B cell involvement in CD4 T cell subsets maturation, including B cell-dependent expansion of the naïve Treg TCR repertoire that enables better control of self-reactive T cells.

## Introduction

Classical functions of B lymphocytes include antigen presentation, antibody secretion, co-stimulation of CD4^+^ T cells, and crosstalk with innate and adaptive T cells (1). However, the role of B cells in the maturation of T cell subsets is not fully understood. Certain B cell subsets have been shown to be involved in the negative selection of T cells in the thymus, suggesting a role for B cells in the induction and maintenance of self-tolerance (2–4). The thymic population of B cells constitutes approximately 0.1–0.3% of thymocytes—comparable to the number of dendritic cells in the thymus (3). It is highly likely that thymic B cells, including those migrating from the periphery (5) involved in the formation of the T cell receptor (TCR) repertoire of naïve CD4^+^ T cells (3, 4). Further interaction of B and T cells could shape the TCR repertoire of both naïve and effector memory T cells in the periphery. Most studies of TCR repertoires in immune pathologies have focused on T cell deficiencies, such as severe combined immunodeficiency (SCID), Omenn’s syndrome, and Wiskott-Aldrich syndrome (6). A considerable decrease in the diversity of the total TCR repertoire, a biased CD4/CD8 ratio, and weak proliferative activity of T cells in response to antigenic stimulation have all been reported for these diseases (6, 7). X-linked agammaglobulinemia (XLA) is a primary immunodeficiency disorder characterized by low levels or absence of immunoglobulins and mature B cells. The pathogenesis of XLA is associated with a loss-of-function mutation in a single gene, *Btk* (Bruton’s tyrosine kinase), resulting in the arrest of B cell differentiation in the bone marrow at the pre-B cell stage. This makes XLA a classical single-factor model to explore the impact of B cell deficiency on T cell immunity. XLA remains understudied in terms of changes in the T cell compartment of the adaptive immune system, but previous studies have demonstrated V gene segment usage differences and increased CDR3 sharing in TCR repertoires for the bulk T cell population in XLA patients compared to healthy donors (8).

We have explored the TCR repertoire of naïve and memory CD4^+^ and CD8^+^ T lymphocytes as well as several functional naïve CD4^+^ (nCD4^+^) subsets in XLA and age-matched healthy young donors. The heterogeneous naïve CD4^+^ population includes naïve T_regs_ and recent thymic emigrants (RTEs), which represent T cell subsets with rather distinct intrinsic properties (9, 10). Before post-thymic selection, the TCR repertoire of RTEs is relatively enriched for self-reactive TCRs. Self-reactive RTEs are predisposed to immune tolerance or anergy, and thus undergo final selection in the periphery. In inflammatory conditions, however, tolerance-prone RTE cells are able to convert into highly competent effector cells (11). T_reg_ cells were originally identified within the CD4^+^CD25^+^ T cell subset, which plays a pivotal role in self-tolerance and prevents autoimmune response (12). Notably, both naïve and effector T_reg_ subsets display TCR repertoire features associated with high self-reactivity and high affinity, which distinguishes T_regs_ from other subsets and indicates their cell fate determination during thymic selection (13, 14).

It has been reported that high rates of infectious diseases in XLA patients (15, 16) can drive early immunosenescence in their T cell populations. To address the potential impact of natural immunosenescence, we completed the analysis with data from healthy elderly repertoires. We observed a reduced proportion of naïve T_reg_ cells among the nCD4^+^ subset, along with high convergence and reduced diversity of naïve T_reg_ TCR repertoires in XLA donors compared to healthy young cohorts. These findings indicate that naïve T_reg_ selection and homeostasis might be impaired in XLA patients. In B cell-deficient mouse models, naïve T_regs_ have been shown to have reduced proliferative capacity and ability to suppress effector cells (17). In parallel, the negative selection of highly autoreactive T cells might be impaired in the absence of a thymic B cell population (3, 4). Several studies in mice have shown that expansion of natural T_reg_ subsets is dependent upon thymic B cells, which can shape T_reg_ TCR repertoires *via* TCR-pMHCII interaction (18, 19). Together with thymic dendritic and epithelial cells, thymic B cells have been shown to induce natural T_reg_ development and proliferation (19).

Our findings support active involvement of the B cell population in naïve T_reg_ selection and homeostasis, and imply a possible link between susceptibility to the development of autoimmune and inflammatory diseases in XLA patients (15, 16) and the proportional reduction of naïve T_reg_ cells, combined with the alteration of naive CD4^+^ and naïve T_reg_ TCR repertoires and naïve T_reg_ transcriptional programs.

## Material and Methods

### Patients and Healthy Donors

XLA and healthy donors were informed of the final use of their blood and signed an informed consent document. The study was approved by the local ethics committee, NRC Institute of Immunology FMBA (Moscow, Russia), protocol 6-1, 09 June 2020, and conducted in accordance with the Declaration of Helsinki. The 10 XLA patients (age 18–36) and a cohort of 15 young donors (age 22–35) and 6 old donors (age 49–83) (**Supplemental Table 1**) were enrolled in the study. All donors were males. Individuals with previously diagnosed cancer or severe autoimmune disease were excluded. The same exclusion criteria were applied to the control cohort. One XLA donor had rheumatoid arthritis affecting both hips, both knees, the right ankle, both wrists, and the metacarpophalangeal joint of the right first finger of the left hand, with ankylosis of the left hip joint, aseptic necrosis of the head of the right femur, and fibrous contracture of the right ankle joint. The other donors had no signs of autoimmune or inflammatory conditions. The baseline treatment for XLA patients included IVIG therapy (0.4 g/kg). We performed immunophenotyping analysis and verified the lack of CD19 expression on XLA PBMCs (**Supplemental Figure 5**).

### Cell Sorting

Peripheral blood (12–20 ml) was collected into a number of EDTA-treated Vacutainer tubes (BD Biosciences, Franklin Lakes). For cell sorting of CD4^+^ and CD8^+^ (defined as CD3^+^ CD4^-^) memory and naïve T cells, RTE, mnCD4+, naïve Treg we used a strategy previously described in Ref (20). Further details see in **Supplemental Methods**. Number of sorted cells for each population see in **Supplemental Tables 2, 3**. All cell populations were collected directly into the RLT buffer (Qiagen) and stored at -70C.

### RNA Isolation and cDNA Library Preparation

Total RNA was isolated using the RNeasy mini kit (Qiagen) according to the manufacturer’s instructions. In all experiments described here, cDNA libraries were obtained using 5’-RACE with unique molecular identifiers (UMI) (20) using Human TCR profiling kit (MiLaboratory LLC). Libraries were sequenced on Illumina NextSeq 500 using 300 cycle reagent kit, paired-end 150 + 150 nt mode.

### Sequencing and Data Analysis

Raw sequencing data was analyzed using MIGEC software v.1.2.9 (21). UMI-labeled TCRβ cDNA molecules were obtained per sample with at least ~1 read per UMI (**Supplemental Tables 2**, **3**). Analysis of the averaged CDR3 characteristics was performed weighted by the abundance of each clonotype. All of the physicochemical characteristics were calculated and averaged for the five amino acid residues located in the middle of CDR3, which are considered to have the highest probability to contact with peptide-MHC complex (20). Further details see in **Supplemental Methods**.

### Statistical Analysis

Statistical analysis was performed on processed datasets with R. Clonotype CDR3 features were calculated for the most frequent V segments extracted from full clone sets to avoid bias related to a particular TRBV segment’s contribution to CDR3 analysis and individual differences in V segment usage. In case of diversity estimation all V segment subsets within the same cell type were down-sampled to the same number of UMI. To exclude potential dependence of clonotype features from the V segment, all features within the same cell type and the same V segment were turned to Z-scores as described in (20). To compare the medians between three groups of samples the Kruskal-Wallis test was used followed by Dunn test with the Benjamini-Hochberg correction for multiple testing, if not mentioned otherwise. To compare the medians between two groups of samples the Wilcoxon rank sum test was applied. Further details see in **Supplemental Methods**. The data is available by PRJNA752656 in the SRA (NCBI).

### RNAseq Library Preparation and Analysis

RNA was isolated using the RNeasy Micro Kit (Qiagen) and analyzed using a Qubit 2.0 Fluorometer (Thermo Fisher Scientific). cDNA libraries were prepared with SMART-Seq v4 Ultra Low Input Kit for Sequencing (Takara Bio). The samples were sequenced using a HiSeq 4000 (75 bp, 10 million average paired reads per sample). Further details see in **Supplemental Methods** and **Supplemental Table 4**.

## Results and Discussion

### Deficiency of Naïve T_reg_ Cells in XLA Patients

**Figure 1:**
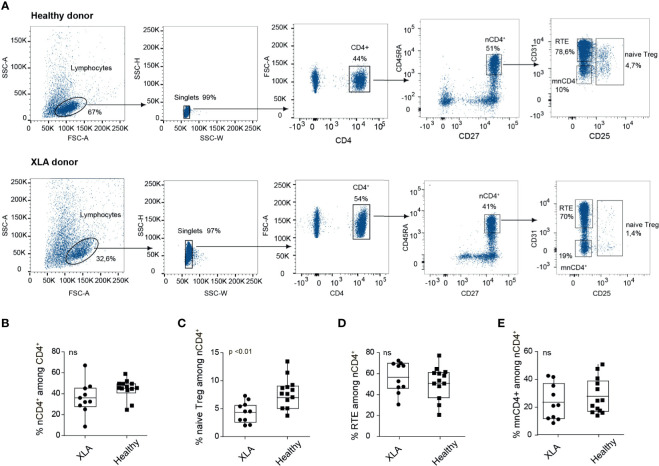
Flow cytometry analysis of CD4^+^ subsets in healthy and XLA donors. **(A)** Gating strategy for cell sorting of nCD4^+^ subsets from healthy (top) and XLA (bottom) young donors, starting from discrimination of lymphocytes by forward (FSC) and side scatter (SSC) profiles and sequential separation of singlets (left), after which CD4^+^ cells were selected (middle). nCD4^+^ were selected as CD45RA^+^CD27^+^, from which RTE, mnCD4^+^and naïve T_reg_ cell subsets were subsequently gated (right). **(B)** Percentage of nCD4^+^ cells among the CD4^+^ subset and of **(C)** naïve T_reg_, **(D)** RTE, and **(E)** mnCD4^+^ among the nCD4^+^ population in XLA (N = 10) and healthy (N = 13) donors. *p*-values were calculated with the Wilcoxon rank-sum test. NS, non significant.

We first assessed the relative abundance of naïve T cells overall as well as distinct naïve T cell subsets in PBMCs collected from seven young (18–36 years old) male XLA patients and five healthy sex- and age-matched donors (**Supplemental Table 1**, **Figure 1**, **Supplemental Figure 1A**). The percentages of naïve CD4^+^ T cells were comparable for the XLA and healthy cohorts (**Figure 1B**). The nCD4^+^ lymphocyte population was subdivided into naïve T_reg_ (CD4^+^CD27^+^CD45RA^+^CD25^+^), mature naïve CD4^+^ (mnCD4^+^; CD4^+^CD27^+^CD45RA^+^CD25^-^CD31^-^), and CD31^+^ cells enriched with recent thymic emigrants (RTE; CD4^+^CD27^+^CD45RA^+^CD25^-^ CD31^+^) (20). Notably, the proportion of naïve T_regs_ among nCD4^+^ T cells was prominently decreased in the XLA cohort (**Figure 1C**) in contrast to RTEs and mnCD4^+^ cells (**Figures 1D, E**). Low T_reg_ counts were previously reported in PBMCs from children with XLA (22) and in spleen of the B cell deficient mice (17). Our data indicate that in the condition associated with primary B cell deficiency, the generation of T_reg_ subsets may be undermined at the level of naïve T_reg_ production.

### Altered TCR Repertoires of Naïve and Memory T Cells

Several studies (23, 24) have demonstrated that the naïve T cell subset is not meaningfully affected at the cellular level in XLA patients. However, the impact of a lack of B cells on TCR repertoire selection has not been deeply explored. To investigate this, we performed unique molecular identifier (UMI)-based 5’-RACE TCRβ profiling, and compared repertoire characteristics for the sorted naïve and memory CD4^+^ and CD8^+^ T cells of young XLA patients *versus* young and old (49-83 y.o.) healthy donors (**Supplemental Figures 1B, C** and **Supplemental Table 2**). To increase the statistical power of analysis, we separately compared diversity metrics for the dominant TRBV segments after down-sampling to an equal number of UMI-labeled TCRβ cDNA molecules, as described in (20).

**Figure 2:**
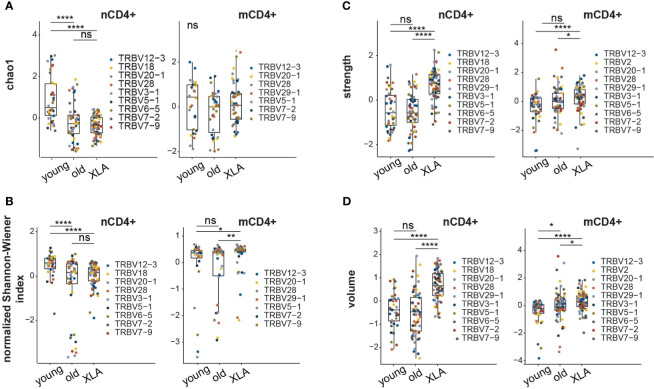
Characteristics of memory and naïve CD4^+^ TCR repertoires in XLA and healthy donors. Analysis of repertoire diversity using the **(A)** Chao1 index and **(B)** normalized Shannon-Wiener index. **(C)** Clonotype interaction strength, as represented by the fraction of hydrophobic and aromatic amino acids, and **(D)** CDR3β volume, based on bulky amino acids, in the central region of CDR3 from XLA (N = 7), and healthy young (N = 6) or old (N = 6) donors. All analyzed features were calculated for the most abundant V-segments extracted from full clonotype datasets. For diversity analysis, all segment sets were down-sampled to 1,000 randomly-chosen UMI-labeled TCRβ cDNA molecules. Only segments with a sufficient number of UMIs were included. To exclude potential dependence of features from the V segment, all features within the same cell type and the same V segment were turned to Z-scores. Differences between groups were examined using the Kruskal-Wallis test followed by the Dunn test with the Benjamini-Hochberg correction. *p < 0.05, **p < 0.01, ****p < 0.0001, ns, non significant.

We assessed normalized diversity metrics, which estimate the evenness of clonotype frequency distribution (*i.e.*, normalized Shannon–Wiener) and the richness of the repertoire based on the number of clonotypes that occur once or twice (*i.e.* Chao1). We observed a decline in Chao1 and normalized Shannon-Wiener diversity values for the nCD4^+^ TCRβ repertoires of young XLA patients compared to sex- and age-matched healthy donors (**Figures 2A, B**). In this respect, the naïve CD4^+^ TCR repertoire of XLA patients reflected a pattern observed in the repertoires of elderly individuals (**Figures 2A, B**). Similar changes in the elderly cohort were previously linked to thymic involution and continuous and biased peripheral proliferation of naïve T cells in the course of cell niche replenishment at the periphery (20, 25). The decrease in TCR diversity for XLA nCD8^+^ cells was less prominent (**Supplemental Figures 2A, B**).

In contrast to the naïve TCR repertoires, we observed more evenly distributed clonotype frequencies in the memory CD4^+^ repertoires of XLA patients compared to matched healthy donors (**Figure 2B**). Previous studies in B cell-deficient mice (26, 27) and clinical data from XLA patients (23) have shown that B cells might be required for appropriate CD4^+^ activation and the generation and maintenance of pathogen-specific memory T cells. In mice, a lack of B cells results in the deeper depletion of antigen-activated CD4^+^ T cells at the contraction phase, followed by the generation of a smaller number of antigen-specific CD4^+^ memory T cells (28). Significant reduction of CD4^+^ memory T cell counts has also been previously reported in XLA donors (23). Thus, the lack of antigen-specific B cell support could contribute to the diminished T cell memory formation in XLA patients. That being said, other evidence indicates that antigen-specific memory T cells can be detected years after immunization of XLA patients, similarly to healthy cohorts (29).

Next, we assessed the physicochemical properties of the amino acid residues forming the central part of the CDR3 (**Figures 2C, D**; **Supplemental Figures 2C, D**, **Figure S3**), which primarily interacts with the antigenic peptide within pMHC complexes (20). On average, nCD8^+^ and nCD4^+^ CDR3β repertoires of XLA patients contained a higher proportion of “strongly interacting” hydrophobic and aromatic amino acids (F, I, L, M, V, W, and Y; **Figure 2C** and **Supplemental Figure 2C**) and bulky amino acid residues (**Figure 2D** and **Supplemental Figure 2D**) compared to matched healthy donors. In our previous experience, the average number of strongly interacting amino acid residues is a basic characteristic determining the average features of the CDR3β repertoire (20, 30, 31). Large numbers of strongly-interacting amino acids may be interpreted as higher average affinity and potentially also increased cross-reactivity of a TCR repertoire (32). The observed increased “strength” in nCD8^+^ and nCD4^+^ repertoires of XLA patients could therefore result from increased competition between naïve T cells for tonic signaling in the absence of antigen-presenting B cells (33). Notably, the difference in the number of strongly-interacting amino acids was more prominent for the nCD4^+^ T cells compared to the memory CD4^+^ (mCD4^+^)T cell subset. There was no significant difference in CDR3 length or insertion size among the naïve or memory CD4^+^ or CD8^+^ T cell subsets (**Supplemental Figure 3**).

### Distinct Properties of Naive CD4^+^ T Cell Subsets

**Figure 3:**
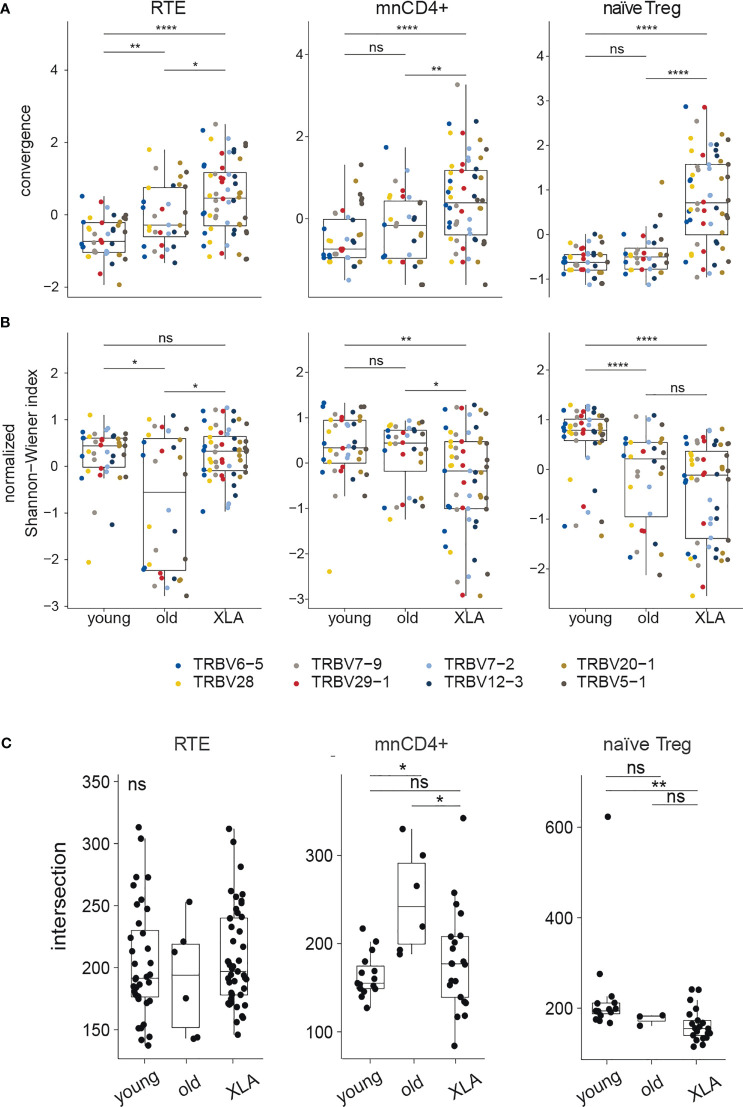
Diversity and convergence of TCRβ repertoires of naïve CD4^+^ subsets in healthy donors and patients with XLA. Analysis of **(A)** convergence and **(B)** normalized Shannon-Wiener index in nCD4^+^ subsets (RTE, mnCD4^+^, naïve T_reg_) in young and old healthy donors and in young XLA patients. Values ​​were obtained for highly abundant TRBV gene segment groups and normalized to mean deviation. For the RTEs, the top 1,524 UMI-labelled sequences were analyzed for every TRBV segment. Donors Y13 and X10 were excluded because of insufficient coverage; this analysis included 8 young healthy donors, 4 old healthy donors, and 6 XLA donors. For the mnCD4^+^, 1,063 UMI -labelled sequences were analyzed for every TRBV segment. Donors Y8, Y13, and X10 were excluded, and this analysis included 7 young healthy donors, 4 old healthy donors, and 6 XLA donors. For T_reg_, 953 UMI-labelled sequences were analyzed for every TRBV segment. Donors X8, X9, X10, Y8, Y10, Y11 were excluded due to insufficient coverage, and this analysis included 6 young healthy donors, 4 old healthy donors, and 4 XLA donors. Significance of median differences was evaluated by Dunn test. The false discovery rate was controlled using the Benjamini-Hochberg adjustment. *p < 0.05, **p < 0.01, ****p < 0.0001, ns, non significant. **(C)** Shows the number of shared public clonotypes between pairs of samples based on CDR3 amino acid sequences, from the 13,000 most abundant clonotypes in each cell subpopulation (for young donors, N = 9 for RTE and mnCD4^+^, and N = 6 for naïve T_reg_; for old donors, N = 4 for RTE and mnCD4^+^, and N = 3 for naïve T_reg_; for XLA patients, N = 10 for RTE and mnCD4^+^, and N = 7 for naïve T_reg_). All samples were down-sampled to the same number of UMIs (23,000 mnCD4^+^, 24,000 RTE, 31,000 naïve T_reg_). NS, non significant.

Heterogeneous mnCD4^+^, RTE, and naïve T_reg_ lymphocyte populations are maintained on the periphery through different mechanisms, and contribute differently to the cumulative landscape repertoire of naïve CD4^+^ Т lymphocytes (20, 34). To explore the impact of each naïve CD4^+^ subset, we analyzed TCR repertoires of sorted mnCD4^+^, RTE, and naïve T_reg_ cell fractions from XLA patients and healthy donors (**Figure 3**). At least 5,000, 28,000, and 13,589 cells were sorted for naïve T_reg_, mnCD4^+^, and RTE subsets, respectively (**Supplemental Table 3**), and UMI-based analysis was performed as above.

For the elderly donors, and to a higher extent for the XLA patients, we observed increased convergence as measured by the relative number of distinct nucleotide sequence variants for each amino acid CDR3 sequence. This effect was observed for all subsets but was more prominent for the naive T_reg_ cells (**Figure 3A**). The latter subset was also characterized by increased clonality in XLA patients, reflected by a decreased normalized Shannon-Wiener index (**Figure 3B**) but a smaller proportion of shared public clonotypes (**Figure 3C**). Such features of XLA patients’ naïve T_reg_ cells may indicate more focused production and peripheral proliferation with narrowed antigenic specificities in an individual MHCII context. This could be a possible consequence of the lack of B cells presenting a wide range of self and commensal antigens both in the thymus and on the periphery for naïve T_reg_ positive selection and further tonic signaling, which is strongly required for T_reg_ homeostasis (35). Altogether, the decline in the naïve T_reg_ proportion among nCD4^+^ cells (**Figure 1C**), increased repertoire convergence (**Figure 3A**) and decreased diversity suggest impaired thymic T_reg_ selection in XLA patients, accompanied by biased peripheral proliferation.

**Figure 4:**
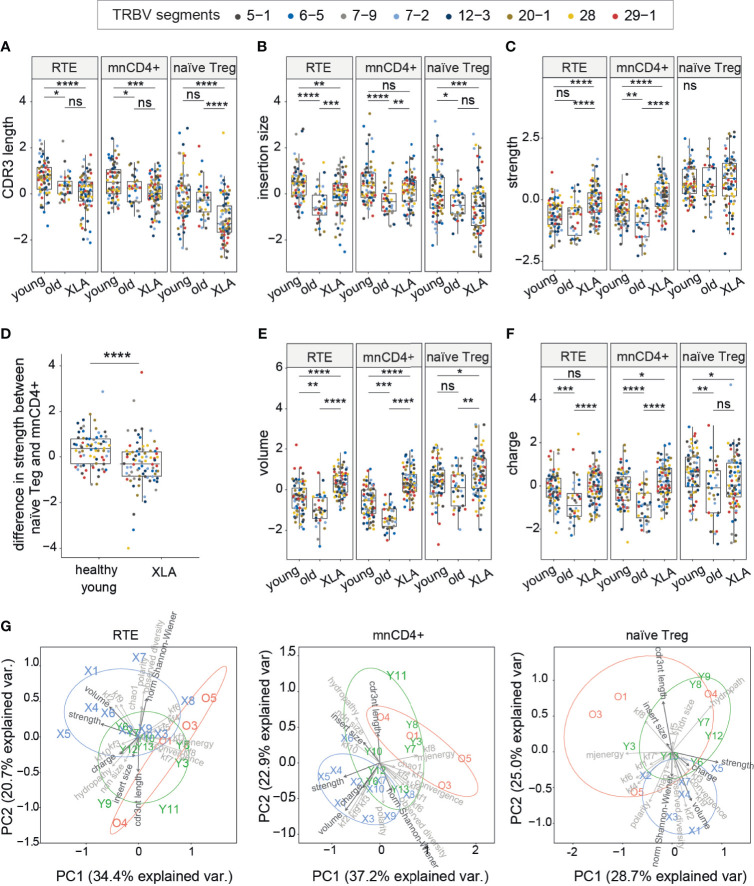
CDR3 characteristics of TCRβ repertoires of naïve CD4^+^ subsets in healthy donors and XLA patients. **(A)** CDR3β length, **(B)** added nucleotides in CDR3β for the most abundant V segments for full clonotype datasets, **(C)** estimated average number of strongly-binding amino acids in the central region of CDR3 for nCD4^+^ populations in XLA and healthy donors. **(D)** The difference in CDR3β interaction strength between naïve T_reg_ and mnCD4^+^ cell repertoires. **(E)** Estimated average number of bulky amino acids, and **(F)** charged amino acids in the central region of CDR3 for nCD4^+^ populations from XLA and healthy young or old donors. The number of healthy young, old and XLA donors was 9, 4 and 10 respectively. For panels **(A–F)**, statistical significance of median differences was evaluated by Dunn test controlled using the Benjamini-Hochberg adjustment. Only segments with a sufficient number of UMIs were included. To exclude potential dependence of features from the V segment, all features within the same cell type and the same V segment were turned to Z-scores. Differences between groups were assessed using the Kruskal-Wallis test followed by Dunn test, with the Benjamini-Hochberg stepwise adjustment. *p < 0.05, **p < 0.01, ***p < 0.001. **(G)** Principal component analysis (PCA) of nCD4^+^ subsets based on 23 TCR repertoire characteristics. PC1 is the first principal component, PC2 is the second. Each dot represents an individual TCR repertoire sample. Arrows in bold show properties from A–F and normalized Shannon-Wiener index. NS, non significant.

The average CDR3β length was shorter in all nCD4^+^ T cell subsets (mnCD4^+^, RTE and naïve T_reg_ cells) of XLA individuals compared to healthy young donors (**Figure 4A**). Additionally, the average number of random nucleotides inserted between Vβ-Dβ and Dβ-Jβ segments in CDR3β was smaller in RTEs and naïve T_reg_ cells (**Figure 4B**). It indicated limited capacity for conformational changes of CDR3β in XLA nCD4^+^ repertoires, potentially reflecting more stringent selection. Shortening of CDR3β length in naïve conventional repertoires also occurred in aged healthy individuals (**Figures 4A, B**) (20). In this respect, the repertoires of the XLA cohort resembled those of elderly individuals.

We next evaluated the physicochemical landscape of TCR repertoires for the nCD4^+^ T cell subsets. The amino acid properties of XLA CDR3β differed substantially from age-matched healthy donors, although the extent of the differences depended on the cell type (**Figures 4C–F**). In particular, the CDR3β repertoires of both RTE and mnCD4^+^ subsets from XLA patients were characterized by increased numbers of strongly interacting amino acid residues (**Figure 4C**), supporting the results obtained for the entire pool of nCD4^+^ T cells (**Figure 2C**). Thus, the difference in strength between mnCD4^+^ cells and naïve T_reg_ cells was decreased in XLA individuals (**Figure 4D**) potentially reducing the average capacity of T_regs_ to suppress activated conventional CD4^+^ T cells. High-affinity TCRs allow T_reg_ cells to compete efficiently and in an antigen-specific fashion with conventional T cells for binding to peptide-MHC complexes presented by APCs (36, 37). Recent data have demonstrated that TCR-specific interactions may be one of the possible suppression mechanisms exerted by T_regs_ (37–39).

Repertoires from all XLA nCD4^+^ subsets differed from their counterparts in young healthy individuals in having an increased average number of bulky amino acid residues in the central region of CDR3β (**Figure 4E**). Additionally, mnCD4^+^ T cells have a more charged CDR3β region in XLA patients than in healthy donors (**Figure 4F**). In contrast, naïve T_regs_ in this group have decreased mean charge within the CDR3β region. The abovementioned shortening of CDR3β might be partially compensated at the expense of a high frequency of bulky amino acid residues within CDR3β in the XLA patient repertoires. Notably, we have shown previously that CDR3β domains from mnCD4^+^ T cells tend to have smaller numbers of strongly interacting and bulky amino acid residues in aged individuals (20), a finding that we have confirmed here (**Figure 4**). However, we observed the opposite in XLA patients, suggesting an association with B cell deficiency rather than general immunosenescence. In other words, the differences in the nCD4^+^ TCR repertoire indicate that the specific process of T cell selection—but not the early exhaustion of naïve T cell pools by pathogen burden—triggers these differences in XLA patients.

We next applied principal component analysis to the nCD4+ TCRβ repertoires of young healthy donors and XLA patients that revealed major contributing parameters, including the CDR3 length and average number of bulky amino acid residues, and illustrated the profound divergence of CDR3β repertoire characteristics between healthy donors and XLA patients (**Figure 4G**).

### Perturbation of Naïve T_reg_ Programs

**Figure 5:**
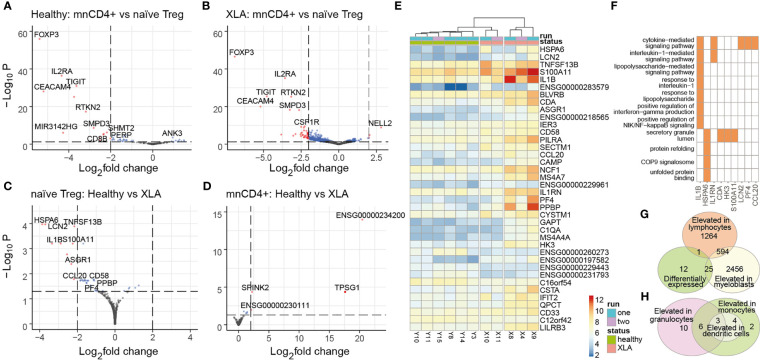
Gene expression analysis in naïve T_regs_. Differential gene expression analysis of naïve T_regs_ and mnCD4^+^ subpopulations in healthy donors **(A)** and patients with XLA **(B)** was performed using DESeq2. Red and blue dots show differentially expressed genes based on p_adj_< 0.05; red dots indicate transcripts based on absolute value of log2 fold changes >2. Adjusted P values were obtained using the Benjamini-Hochenberg procedure. Shrunken log2 fold changes were calculated using the ashr estimator, with mnCD4^+^ gene expression as numerator and naïve T_reg_ gene expression as denominator. **(C)** Differential gene expression analysis of naïve T_reg_ and **(D)** mnCD4^+^ with the healthy group as numerator and XLA as denominator. Colored dots highlight transcripts overexpressed in XLA naïve T_reg_ (p_adj_< 0.05), red dots mark genes with log2 fold changes >2. **(E)** Heatmap of differentially expressed genes for naïve T_reg_ cells from XLA and healthy donors. Data was prepared by DEseq2 as described in the *Material and Methods* section. **(F)** GO-enriched terms and input genes that are highly expressed in XLA naïve T_reg_. Enrichment analysis was performed by pathfindR package, with 0.05 threshold for p-adj following DESeq analysis. **(G)** Venn diagram of XLA naïve T_reg_ differentially expressed transcripts, showing intersection with genes that are usually expressed in myeloblasts or lymphocytes, or **(H)** intersection with genes usually elevated in granulocytes, dendritic cells, or monocytes according to www.proteinatlas.org.

To get insights on the functional alterations of mnCD4^+^ and naïve T_reg_ cell subsets, we performed transcriptomic analysis of these subsets from XLA and healthy young donors (**Figure 5**). Despite the fact that the mnCD4^+^ cells showed a significant change in the physicochemical properties of their TCR repertoires, we observed no essential changes in their transcriptome (**Figure 5D**).

We evaluated classical T_reg_ signatures of gene expression (*e.g.*, FOXP3, IL2RA, TIGIT, CEACAM4, RTKN2) in the sorted naïve T_reg_ cells in comparison to mnCD4^+^ cells from XLA and healthy donors (**Figures 5A, B**). XLA naïve T_regs_ maintained comparable expression of T_reg_ phenotype-specific genes to healthy donors (**Figure 5C**) as well as T-cell-specific genes (**Supplemental Figure S4**). Functionally distinct T_reg_ subsets that can be identified based on expression of certain transcription factors (*i.e.*, Bcl6, Stat4, Stat3, T-bet, RORC) (40) and chemokine receptors (i.e., CCR6, CXCR3) (41) were also not overrepresented in XLA naïve T_reg_ relative to healthy donors.

Nevertheless, we found several atypical transcriptomic features of XLA naïve T_reg_ in comparison to healthy donors. Among 39 gene transcripts enriched in XLA naïve T_reg_ were genes that are abundantly expressed by antigen-presenting cells (APCs) and myeloid cells, including *IL1b, SECTM1, CD33, LILRB3, PILRA, NCF1*, and *TACI* (**Figures 5E–H**). *TACI*, also known as *TNFRSF13B*, is a receptor for a proliferation-inducing ligand (APRIL) and B cell-activating factor of the tumor necrosis factor family (BAFF) (42), which is predominantly expressed on B cells. Recent evidence indicates that *TACI* expression in T_reg_ cells promotes their survival and proliferation (43), and the upregulation of *TACI* in XLA naïve T_reg_ cells suggests a mechanism of homeostatic naïve T_reg_ expansion in B cell-deficient conditions. Several genes enriched in XLA cell populations (*e.g.*, *SECTM1*, *CD58*) encode co-stimulatory molecules involved in T cell activation (44, 45). *CD58* is widely expressed by both hematopoietic and non-hematopoietic cells, including B lymphocytes (46). The functional outcome of CD58-CD2 interaction in CD4^+^ T cells remains poorly understood, but this interaction with CD2 on NK and effector T cells has been shown to contribute to T cell proliferation and NK cell activation (47). Importantly, it can also induce rapid differentiation to an antigen-specific T_reg_ cell subtype, Tr1, that is characterized by high IL-10 production (48). Thus, *CD58* upregulation in XLA might provide additional positive feedback stimulation of T_reg_ cells.

Interestingly, we also found enrichment of chemokine genes in the XLA naïve T_reg_ transcriptome, including *CCL20, CXCL4* (*PF4*), and *PPBP (CXCL7*) (**Figures 5E, F**). *CXCL4* has been shown to exert Th17 induction in autoimmune diseases (49). Expression of *CCL20* was previously reported in Tfh and Th17 cells and at very low levels in T_regs_, where the latter is mediated synergistically by TGF-β/IL-6 (50).

Our data may indicate that peripheral maturation of XLA naïve T_reg_ cells occur under pro-inflammatory conditions that promote aberrant expression of proinflammatory markers and mediators (*e.g.*, *IL1b*, *CXCL4*, *CCL20* and *S100A11*) (49, 51). Interestingly, we also detected several atypically expressed genes that are normally specific to dendritic cells and granulocytes (**Figures 5G, H**). For example, *IL1b* expression by lymphoid cells is not typical, although it has been described previously for CCR5^+^CD4^+^ T cells (52). Our data assume that enhanced *S100A11* and *IER3* gene expression might be related to *IL1b* levels in XLA patients. *IL1b*-dependent up-regulation of *IER3* has been reported to increase T cell lifespan (53), hinting at possible changes in naïve T_reg_ homeostasis in XLA donors.

## Conclusion

Recent studies on primary immunodeficiency highlight critical gaps in current knowledge about how the TCR repertoire is shaped in the absence of B cells. Our study suggests that one of the most essential impacts of congenital B cell deficiency on the T cell branch of the immune system is on nCD4^+^ T cell subsets, and especially on naïve T_reg_ selection and homeostasis. Specifically, we observed the decline in the naïve T_reg_ proportion among nCD4^+^ cells, increased convergence and clonality of the naïve T_reg_ TCR repertoire, specific differences in the averaged CDR3β characteristics of nCD4^+^ subsets that are distinct from those that accumulate with aging, and some notable alterations in the naïve T_reg_ transcriptome. Collectively, these findings indicate potential alterations in the selection, maturation, and peripheral proliferation of naïve T_regs_ in XLA patients. Further studies with a wider methodical arsenal and larger patient cohorts will be required to confirm our findings and better elucidate the nature of the observed phenomena, but our results indicate that the intimate relationship between B cell and T_reg_ subsets (17) should remain the active focus of current investigations.

## Data Availability Statement

The datasets presented in this study can be found in online repositories. The names of the repository/repositories and accession number(s) can be found below: https://www.ncbi.nlm.nih.gov/sra/, PRJNA752656, PRJNA752868; https://figshare.com/articles/dataset/Na_ve_Regulatory_T_Cell_Subset_Is_Altered_in_X-Linked_Agammaglobulinemia/15128715; https://figshare.com/articles/dataset/Untitled_Item/15131340.

## Ethics Statement

The studies involving human participants were reviewed and approved by NRC Institute of Immunology FMBA (Moscow, Russia), protocol 6-1, 09 June 2020. The patients/participants provided their written informed consent to participate in this study.

## Author Contributions

OB, DC, SL, MK, and IK designed research. TN, SK, VK, EE, DS, IS and EM performed research. PS, KL, VK analyzed data. EL and IM collected patient samples. DC, PS, KL, and OB wrote the paper. All authors contributed to the article and approved the submitted version.

## Funding

The work was supported by grant of the Ministry of Science and Higher Education of the Russian Federation № 075-15-2020-807.

## Conflict of Interest

The authors declare that the research was conducted in the absence of any commercial or financial relationships that could be construed as a potential conflict of interest.

## Publisher’s Note

All claims expressed in this article are solely those of the authors and do not necessarily represent those of their affiliated organizations, or those of the publisher, the editors and the reviewers. Any product that may be evaluated in this article, or claim that may be made by its manufacturer, is not guaranteed or endorsed by the publisher.
