# Supplementary material for: Naïve Regulatory T Cell Subset Is Altered in X-Linked Agammaglobulinemia
Source: Front Immunol. 2021 Aug 19;12:697307. doi: 10.3389/fimmu.2021.697307 (PMC8417104; doi:10.3389/fimmu.2021.697307)
Supplement: Supplementary file 1 [file DataSheet_1.docx]

**Supplementary Material for**

Naive regulatory T cell subset is altered in X-linked agammaglobulinemia.

Shelyakin P.V. et al.

*Corresponding author e-mail: olbritan@gmail.com

This file includes:

Tables S1 to S4

Figures S1 to S3

Supplemental Materials and Methods

Supplemental References

**Supplementary Table S1. Age of donors included in the cell sorting experiments.** There was no significant difference (*p* < 0.43, two sample t-test) in age between healthy young and XLA donors.

| Donor ID | Age | Naive&memory T cell sorting | nCD4 subset sorting | RNA-seq | Status | Mean age for the group |
| --- | --- | --- | --- | --- | --- | --- |
| O1 | 55 | yes | yes | no | healthy old | 65.8 |
| O2 | 75 | yes | no | no | healthy old |  |
| O3 | 83 | yes | yes | no | healthy old |  |
| O4 | 78 | yes | yes | no | healthy old |  |
| O5 | 49 | yes | yes | no | healthy old |  |
| O6 | 55 | yes | no | no | healthy old |  |
| X1 | 18 | yes | yes | no | XLA | 26.9 |
| X2 | 31 | yes | yes | no | XLA |  |
| X3 | 18 | yes | yes | no | XLA |  |
| X4 | 33 | yes | yes | yes | XLA |  |
| X5 | 36 | yes | yes | no | XLA |  |
| X6 | 34 | yes | yes | no | XLA |  |
| X7 | 30 | yes | yes | no | XLA |  |
| X8 | 19 | no | yes | yes | XLA |  |
| X9 | 30 | no | yes | yes | XLA |  |
| X10 | 20 | no | yes | yes | XLA |  |
| Y1 | 31 | yes | yes | no | healthy young | 28.9 |
| Y2 | 35 | yes | no | no | healthy young |  |
| Y3 | 25 | yes | yes | yes | healthy young |  |
| Y4 | 29 | yes | no | no | healthy young |  |
| Y5 | 33 | yes | no | no | healthy young |  |
| Y6 | 29 | no | yes | no | healthy young |  |
| Y7 | 32 | yes | yes | no | healthy young |  |
| Y8 | 26 | no | yes | no | healthy young |  |
| Y9 | 31 | no | yes | no | healthy young |  |
| Y10 | 26 | no | yes | yes | healthy young |  |
| Y11 | 24 | no | yes | yes | healthy young |  |
| Y12 | 30 | no | yes | no | healthy young |  |
| Y13 | 28 | no | yes | no | healthy young |  |
| Y14 | 22 | no | no | yes | healthy young |  |
| Y15 | 33 | no | no | yes | healthy young |  |

**Supplementary Table S2. Sequencing data on TCR repertoires of naive and memory T cell subsets in healthy and XLA donors.**

| Donor ID | Cell type | Status | Number of sorted cells | Total number of paired reads (raw) | Number of UMI (counts) from aligned functional sequences (threshold 1) |
| --- | --- | --- | --- | --- | --- |
| O1 | CD4 memory | healthy old | 300000 | 2330467 | 618654 |
| O1 | CD4 naive | healthy old | 200000 | 2240220 | 572471 |
| O1 | CD8 memory | healthy old | 200000 | 1493828 | 413494 |
| O1 | CD8 naive | healthy old | 200000 | 1390589 | 331903 |
| O2 | CD4 memory | healthy old | 300000 | 2169391 | 407682 |
| O2 | CD4 naive | healthy old | 300000 | 530258 | 96994 |
| O2 | CD8 naive | healthy old | 60000 | 181847 | 28304 |
| O3 | CD4 memory | healthy old | 200000 | 1188282 | 353104 |
| O3 | CD4 naive | healthy old | 190000 | 2191332 | 576971 |
| O3 | CD8 memory | healthy old | 75000 | 1024344 | 269138 |
| O3 | CD8 naive | healthy old | 12000 | 182559 | 60998 |
| O4 | CD4 memory | healthy old | 100000 | 1533425 | 312381 |
| O4 | CD4 naive | healthy old | 58400 | 1583383 | 142639 |
| O4 | CD8 memory | healthy old | 100000 | 639473 | 142334 |
| O4 | CD8 naive | healthy old | 9250 | 403449 | 40438 |
| O5 | CD4 memory | healthy old | 304000 | 1047115 | 369613 |
| O5 | CD4 naive | healthy old | 300000 | 1778966 | 490267 |
| O5 | CD8 memory | healthy old | 213000 | 1253241 | 400856 |
| O5 | CD8 naive | healthy old | 105000 | 1102012 | 393467 |
| O6 | CD4 memory | healthy old | 300000 | 163703 | 15437 |
| O6 | CD4 naive | healthy old | 150000 | 359720 | 25716 |
| O6 | CD8 memory | healthy old | 300000 | 343359 | 39798 |
| O6 | CD8 naive | healthy old | 100000 | 288934 | 17172 |
| X1 | CD4 memory | XLA | 190000 | 1458318 | 203967 |
| X1 | CD4 naive | XLA | 200000 | 1420250 | 237168 |
| X1 | CD8 memory | XLA | 45000 | 441958 | 65578 |
| X1 | CD8 naive | XLA | 47000 | 499610 | 67734 |
| X2 | CD4 memory | XLA | 300000 | 2328990 | 667082 |
| X2 | CD4 naive | XLA | 90000 | 1332053 | 370952 |
| X2 | CD8 memory | XLA | 300000 | 1788389 | 338321 |
| X2 | CD8 naive | XLA | 86000 | 534476 | 107238 |
| X3 | CD4 memory | XLA | 170000 | 1579727 | 211647 |
| X3 | CD4 naive | XLA | 180000 | 1034922 | 228737 |
| X3 | CD8 memory | XLA | 300000 | 1391606 | 212678 |
| X3 | CD8 naive | XLA | 160000 | 1363199 | 185751 |
| X4 | CD4 memory | XLA | 205900 | 1741321 | 324635 |
| X4 | CD4 naive | XLA | 203900 | 1976928 | 353453 |
| X4 | CD8 memory | XLA | 58900 | 1015104 | 284765 |
| X4 | CD8 naive | XLA | 135200 | 1877057 | 430777 |
| X5 | CD4 memory | XLA | 209400 | 1518782 | 434814 |
| X5 | CD4 naive | XLA | 164700 | 1644108 | 380478 |
| X5 | CD8 memory | XLA | 207600 | 1121537 | 351410 |
| X5 | CD8 naive | XLA | 205200 | 1203754 | 341509 |
| X6 | CD4 memory | XLA | 183000 | 824388 | 152747 |
| X6 | CD4 naive | XLA | 200000 | 989675 | 167846 |
| X6 | CD8 memory | XLA | 127000 | 481130 | 101789 |
| X6 | CD8 naive | XLA | 60000 | 592415 | 75266 |
| X7 | CD4 memory | XLA | 200000 | 2133880 | 477699 |
| X7 | CD4 naive | XLA | 200000 | 2132147 | 406435 |
| X7 | CD8 memory | XLA | 300000 | 2505246 | 620529 |
| X7 | CD8 naive | XLA | 131000 | 1691171 | 290494 |
| Y2 | CD4 memory | healthy young | 200000 | 141614 | 37128 |
| Y2 | CD4 naive | healthy young | 200000 | 1172647 | 138035 |
| Y2 | CD8 memory | healthy young | 200000 | 63574 | 20640 |
| Y2 | CD8 naive | healthy young | 200000 | 972143 | 94326 |
| Y3 | CD4 memory | healthy young | 100000 | 362098 | 133453 |
| Y3 | CD4 naive | healthy young | 200000 | 1407700 | 242047 |
| Y3 | CD8 memory | healthy young | 100000 | 498459 | 57120 |
| Y3 | CD8 naive | healthy young | 100000 | 439871 | 181693 |
| Y4 | CD4 memory | healthy young | 200000 | 419978 | 65785 |
| Y4 | CD4 naive | healthy young | 150000 | 446586 | 72364 |
| Y4 | CD8 memory | healthy young | 200000 | 141131 | 31167 |
| Y4 | CD8 naive | healthy young | 200000 | 347094 | 48649 |
| Y5 | CD4 memory | healthy young | 300000 | 698076 | 100479 |
| Y5 | CD4 naive | healthy young | 300000 | 1009843 | 118602 |
| Y5 | CD8 memory | healthy young | 300000 | 491595 | 65599 |
| Y5 | CD8 naive | healthy young | 300000 | 428576 | 56365 |

**Supplementary Table S3. Sequencing data on TCR repertoires of nCD4^+^ T cell subsets in healthy and XLA donors.**

| Donor ID | Cell type | Status | Number of sorted cells | Total number of paired reads | Number of UMI (counts) from aligned functional sequences (threshold 1) | Number of UMI (counts) from aligned functional sequences (threshold 2) |
| --- | --- | --- | --- | --- | --- | --- |
| O1 | MN CD4+ | healthy old | 189000 | 2043345 | 321349 | 156952 |
| O3 | MN CD4+ | healthy old | 100000 | 2906904 | 577411 | 212809 |
| O4 | MN CD4+ | healthy old | 28072 | 793612 | 86867 | 35893 |
| O5 | MN CD4+ | healthy old | 323000 | 2622294 | 646992 | 223748 |
| Y6 | MN CD4+ | healthy young | 50000 | 470985 | 53605 | 38488 |
| Y7 | MN CD4+ | healthy young | 144000 | 1338766 | 83156 | 62492 |
| Y3 | MN CD4+ | healthy young | 62574 | 2549767 | 209207 | 114159 |
| Y8 | MN CD4+ | healthy young | 30000 | 495295 | 24333 | 22407 |
| Y9 | MN CD4+ | healthy young | 50000 | 593328 | 67031 | 35586 |
| Y10 | MN CD4+ | healthy young | 83000 | 1526602 | 68149 | 63072 |
| Y11 | MN CD4+ | healthy young | 72000 | 1396886 | 47505 | 43084 |
| Y12 | MN CD4+ | healthy young | 100000 | 736639 | 66325 | 42160 |
| Y13 | MN CD4+ | healthy young | 100000 | 457562 | 42486 | 38619 |
| X1 | MN CD4+ | XLA | 78000 | 4260597 | 863985 | 247626 |
| X2 | MN CD4+ | XLA | 51000 | 3022054 | 755989 | 190780 |
| X3 | MN CD4+ | XLA | 33000 | 1093429 | 271215 | 76836 |
| X8 | MN CD4+ | XLA | 100000 | 1639200 | 74820 | 68849 |
| X4 | MN CD4+ | XLA | 194800 | 1223852 | 789834 | 204758 |
| X5 | MN CD4+ | XLA | 49100 | 598713 | 359602 | 67603 |
| X6 | MN CD4+ | XLA | 190000 | 3863017 | 330943 | 151587 |
| X7 | MN CD4+ | XLA | 100000 | 2128540 | 206551 | 144939 |
| X9 | MN CD4+ | XLA | 99000 | 1469998 | 48293 | 43882 |
| X10 | MN CD4+ | XLA | 94000 | 1998287 | 97035 | 89065 |
| O1 | naive Treg | healthy old | 10700 | 310625 | 80589 | 17908 |
| O3 | naive Treg | healthy old | 12000 | 525318 | 76220 | 19392 |
| O4 | naive Treg | healthy old | 16853 | 1195470 | 93608 | 24560 |
| O5 | naive Treg | healthy old | 6100 | 229621 | 80739 | 26278 |
| Y6 | naive Treg | healthy young | 14000 | 269023 | 35242 | 12415 |
| Y7 | naive Treg | healthy young | 20200 | 257518 | 32578 | 12513 |
| Y3 | naive Treg | healthy young | 27209 | 1490885 | 98480 | 36160 |
| Y8 | naive Treg | healthy young | 22000 | 412909 | 9400 | 8239 |
| Y9 | naive Treg | healthy young | 12000 | 650526 | 79602 | 18014 |
| Y10 | naive Treg | healthy young | 17000 | 221675 | 6453 | 5844 |
| Y11 | naive Treg | healthy young | 19000 | 290758 | 6351 | 5680 |
| Y12 | naive Treg | healthy young | 24000 | 195519 | 41874 | 11837 |
| Y13 | naive Treg | healthy young | 100000 | 940613 | 62040 | 45507 |
| X1 | naive Treg | XLA | 35600 | 1531419 | 359692 | 78057 |
| X2 | naive Treg | XLA | 8400 | 446788 | 77355 | 24007 |
| X3 | naive Treg | XLA | 8000 | 466671 | 115627 | 23929 |
| X8 | naive Treg | XLA | 8000 | 150551 | 3900 | 3530 |
| X4 | naive Treg | XLA | 28200 | 3057744 | 350508 | 69723 |
| X11 | naive Treg | XLA | 22300 | 248726 | 136902 | 18771 |
| X6 | naive Treg | XLA | 12000 | 1096572 | 54251 | 10968 |
| X7 | naive Treg | XLA | 42000 | 1636683 | 284577 | 80358 |
| X9 | naive Treg | XLA | 6000 | 79844 | 1448 | 1293 |
| X10 | naive Treg | XLA | 5000 | 90279 | 1920 | 1628 |
| O1 | RTE | healthy old | 89000 | 889597 | 107969 | 73052 |
| O3 | RTE | healthy old | 38000 | 1437936 | 311263 | 87857 |
| O4 | RTE | healthy old | 13589 | 771670 | 65447 | 15895 |
| O5 | RTE | healthy old | 278000 | 2181649 | 737941 | 147979 |
| Y6 | RTE | healthy young | 50000 | 1012540 | 78115 | 43684 |
| Y7 | RTE | healthy young | 101600 | 1352037 | 110401 | 75998 |
| Y3 | RTE | healthy young | 200000 | 4833735 | 361526 | 282498 |
| Y8 | RTE | healthy young | 65000 | 1912640 | 51533 | 46717 |
| Y9 | RTE | healthy young | 50000 | 2158803 | 113779 | 79069 |
| Y10 | RTE | healthy young | 100000 | 1719074 | 62098 | 57269 |
| Y11 | RTE | healthy young | 100000 | 2720945 | 68486 | 62140 |
| Y12 | RTE | healthy young | 63000 | 782534 | 85155 | 58059 |
| Y13 | RTE | healthy young | 100000 | 399037 | 25024 | 22643 |
| X1 | RTE | XLA | 100000 | 4824002 | 919183 | 311655 |
| X2 | RTE | XLA | 63000 | 1711852 | 593317 | 124202 |
| X3 | RTE | XLA | 100000 | 5378974 | 888787 | 282102 |
| X8 | RTE | XLA | 100000 | 2111958 | 80849 | 74430 |
| X4 | RTE | XLA | 195900 | 3134087 | 789834 | 199881 |
| X5 | RTE | XLA | 204000 | 1330223 | 860097 | 215747 |
| X6 | RTE | XLA | 300000 | 2711716 | 326189 | 160291 |
| X7 | RTE | XLA | 200000 | 4343520 | 958138 | 285878 |
| X9 | RTE | XLA | 100000 | 2166006 | 68567 | 63244 |
| X10 | RTE | XLA | 100000 | 3112793 | 125698 | 116025 |

**Supplementary Table S4. STAR alignment data on RNA-seq for nCD4^+^ T cell subsets in healthy and XLA donors**

| Donor ID | Cell type | Total number of reads | Uniquely mapped reads % | Reads mapped to multiple loci, % | Reads unmapped, % |
| --- | --- | --- | --- | --- | --- |
| X4 | MN CD4+ | 30632044 | 84,73 | 5,22 | 9,81 |
| X4 | naive Treg | 30029556 | 87,39 | 4,30 | 8,13 |
| X5 | MN CD4+ | 30674684 | 86,26 | 4,61 | 8,91 |
| X5 | naive Treg | 30160220 | 86,01 | 4,54 | 9,25 |
| X8 | MN CD4+ | 39137111 | 84,94 | 4,92 | 9,90 |
| X9 | MN CD4+ | 16772512 | 82,45 | 5,56 | 11,74 |
| X9 | naive Treg | 21909157 | 84,40 | 4,43 | 10,99 |
| X10 | MN CD4+ | 19450031 | 83,86 | 5,27 | 10,58 |
| X10 | naive Treg | 16418419 | 84,59 | 4,19 | 11,05 |
| Y3 | naive Treg | 19779343 | 80,50 | 5,02 | 14,24 |
| Y8 | MN CD4+ | 13781205 | 85,83 | 4,93 | 9,08 |
| Y8 | naive Treg | 18493797 | 86,57 | 3,96 | 9,33 |
| Y10 | MN CD4+ | 18907655 | 86,30 | 4,20 | 9,30 |
| Y10 | naive Treg | 22131898 | 85,53 | 4,39 | 9,89 |
| Y11 | MN CD4+ | 16102186 | 83,47 | 4,78 | 11,53 |
| Y11 | naive Treg | 19841472 | 85,34 | 4,34 | 10,14 |
| Y14 | MN CD4+ | 31151485 | 83,78 | 5,34 | 10,60 |
| Y14 | naive Treg | 31584154 | 85,25 | 4,82 | 9,72 |
| Y15 | MN CD4+ | 21833653 | 87,05 | 4,12 | 8,68 |
| Y15 | naive Treg | 23880769 | 87,24 | 4,56 | 8,02 |


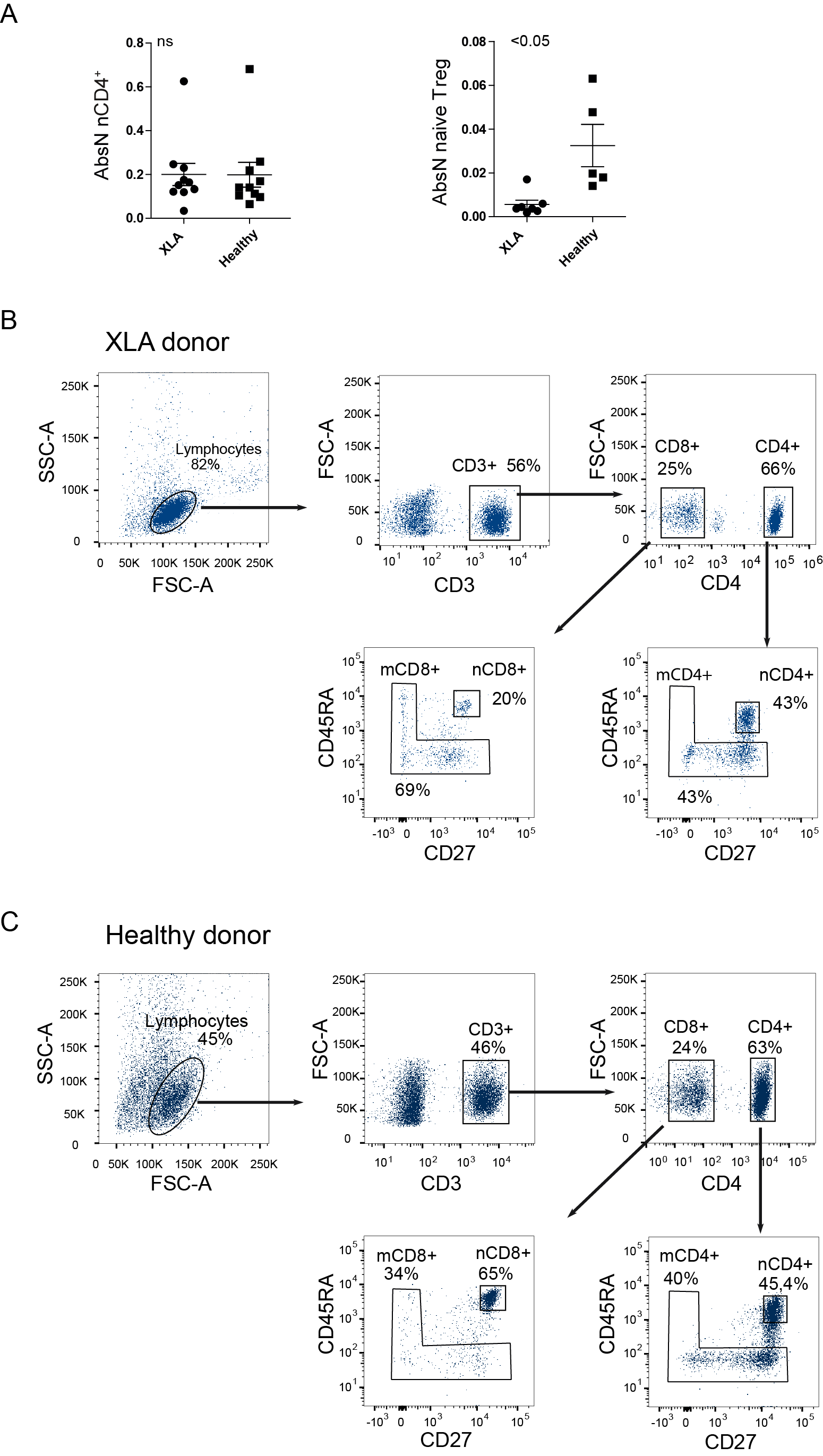


**Supplementary Figure S1. Cell count of nCD4^+^ and naïve T_reg_ cells and gating strategy for cell sorting of naive and memory CD4^+^ and CD8^+^ cells. (A)** Absolute count for nCD4+ and naïve T_reg_ cells (10^3 cells per ul blood). Naive and memory CD4^+^ and CD8^+^ cells for (B) XLA and (C) healthy donors. Lymphocytes were gated based on forward (FSC) and side scatter (SSC) profiles; doublets were excluded in the next step (not shown). Within the singlets, CD3^+^ and (sequentially) CD8^+^ and CD4^+^ cell subsets were discriminated. Naive CD4^+^ and CD8^+^ were defined as CD45RA^+^CD27^+^ on the sequential gates.


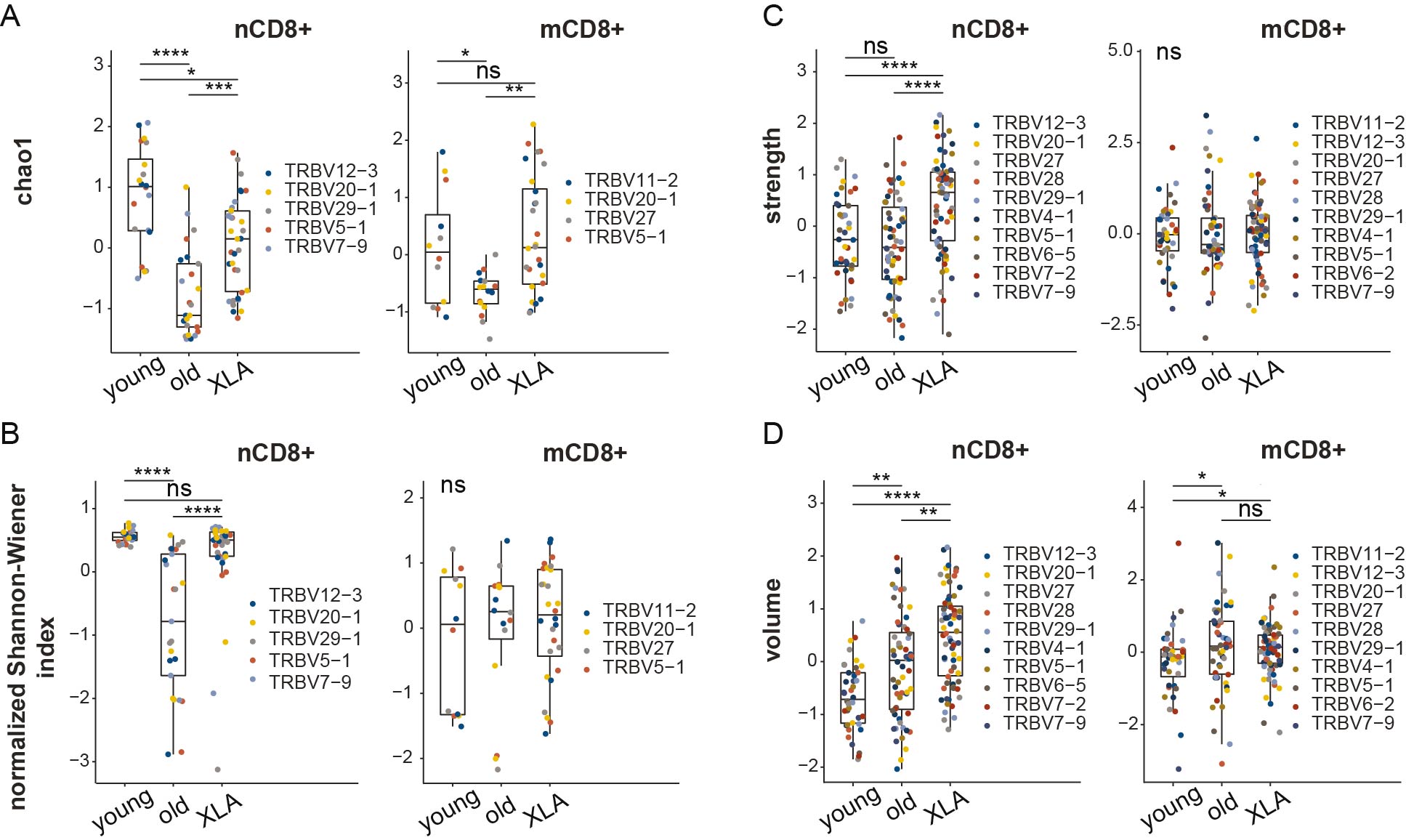


**Supplementary Figure S2**. **Characteristics of memory and naive CD8^+^ TCR repertoires in XLA and healthy donors.** Analysis of repertoire diversity using the (**A**) Chao1 index and (**B**) normalized Shannon-Wiener index. (**C**) Clonotype interaction strength, as represented by the fraction of hydrophobic and aromatic amino acids, and (**D**) clonotype volume, based on bulky amino acids, in the central region of CDR3 from XLA (N = 7), and healthy young (N = 6) or old (N = 6) donors. All analyzed features were calculated for the most abundant V segments extracted from full clonotype datasets. For diversity analysis, all segment sets were down-sampled to 800 (naïve CD8^+^), and 750 (memory CD8^+^) randomly-chosen UMI-labeled TCRβ cDNA molecules. Only segments with a sufficient number of UMIs were included. To exclude potential dependence of features from the V segment, all features within the same cell type and the same V segment were turned to Z-scores. Differences between groups were examined using the Kruskal-Wallis test followed by the Dunn test with the Benjamini-Hochberg correction. *p<0.05, **p<0.01, ***p<0.001, ****p<0.0001.


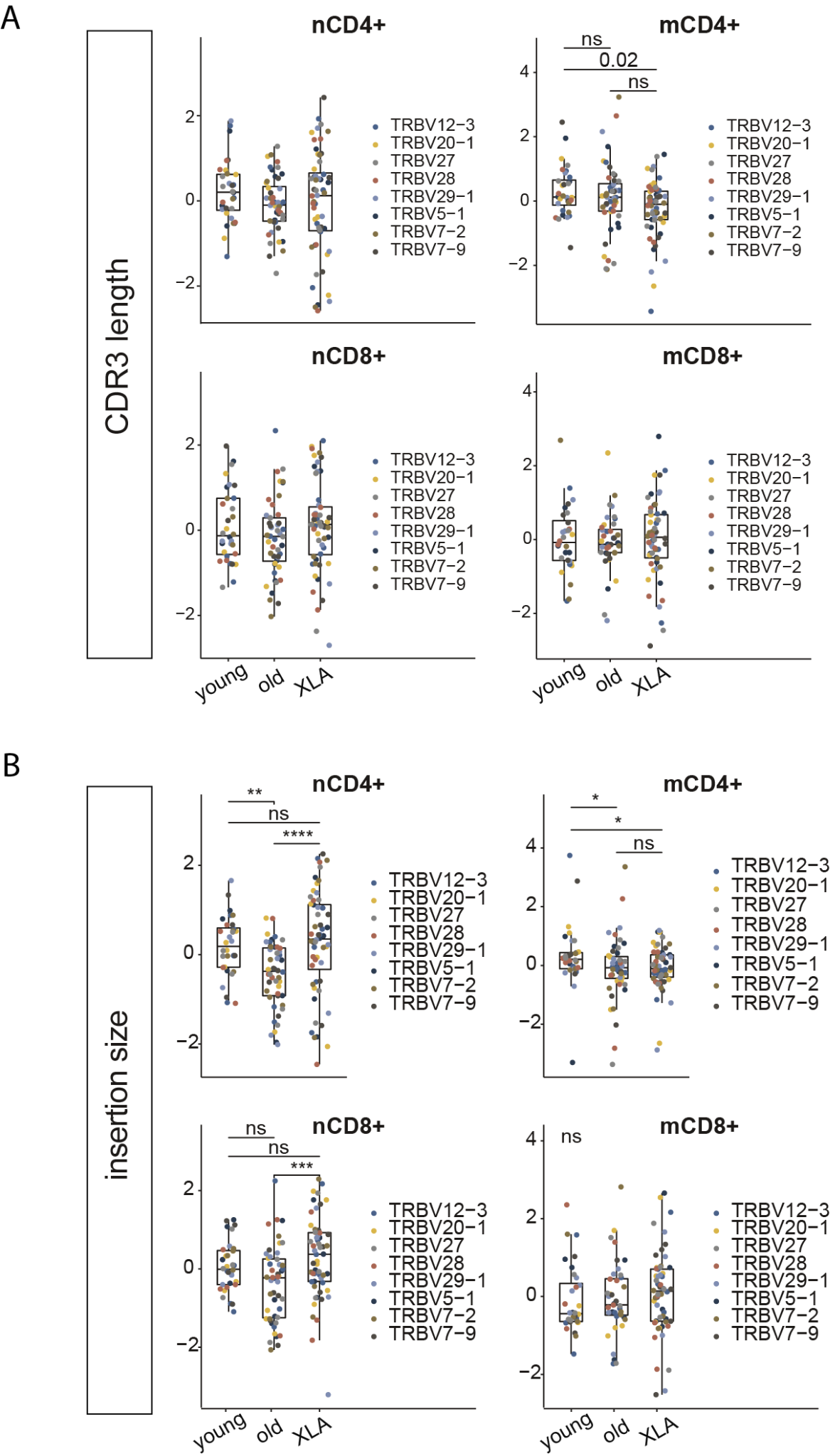


**Supplementary Figure S3.**  **Characteristics of TCR repertoires of CD4^+^ and CD8^+^ cell subsets in XLA and healthy donors.** (A) CDR3β length and (B) added nucleotides in CDR3β were calculated for the most frequent V segments extracted from full datasets. The number of samples was XLA N=7, young N=6, old N=6. All analyzed features were calculated for the most abundant V segments extracted from full clone sets. For diversity analysis, all segment sets were down-sampled to 1,000 (for naive and memory CD4^+^), 800 (for naïve CD8^+^) and 750 (for memory CD8^+^) randomly chosen UMI-labeled TCRβ beta cDNA molecules. Only segments with enough number of UMI were included in the analysis. To exclude potential dependence of features from the V segment, all features within the same cell type and the same V segment were turned to Z-scores. The difference between groups was examined using the Kruskal-Wallis test followed by the Dunn test with Benjamini-Hochberg correction. *p<0.05, **p<0.01, ***p<0.001, ****p<0.0001.


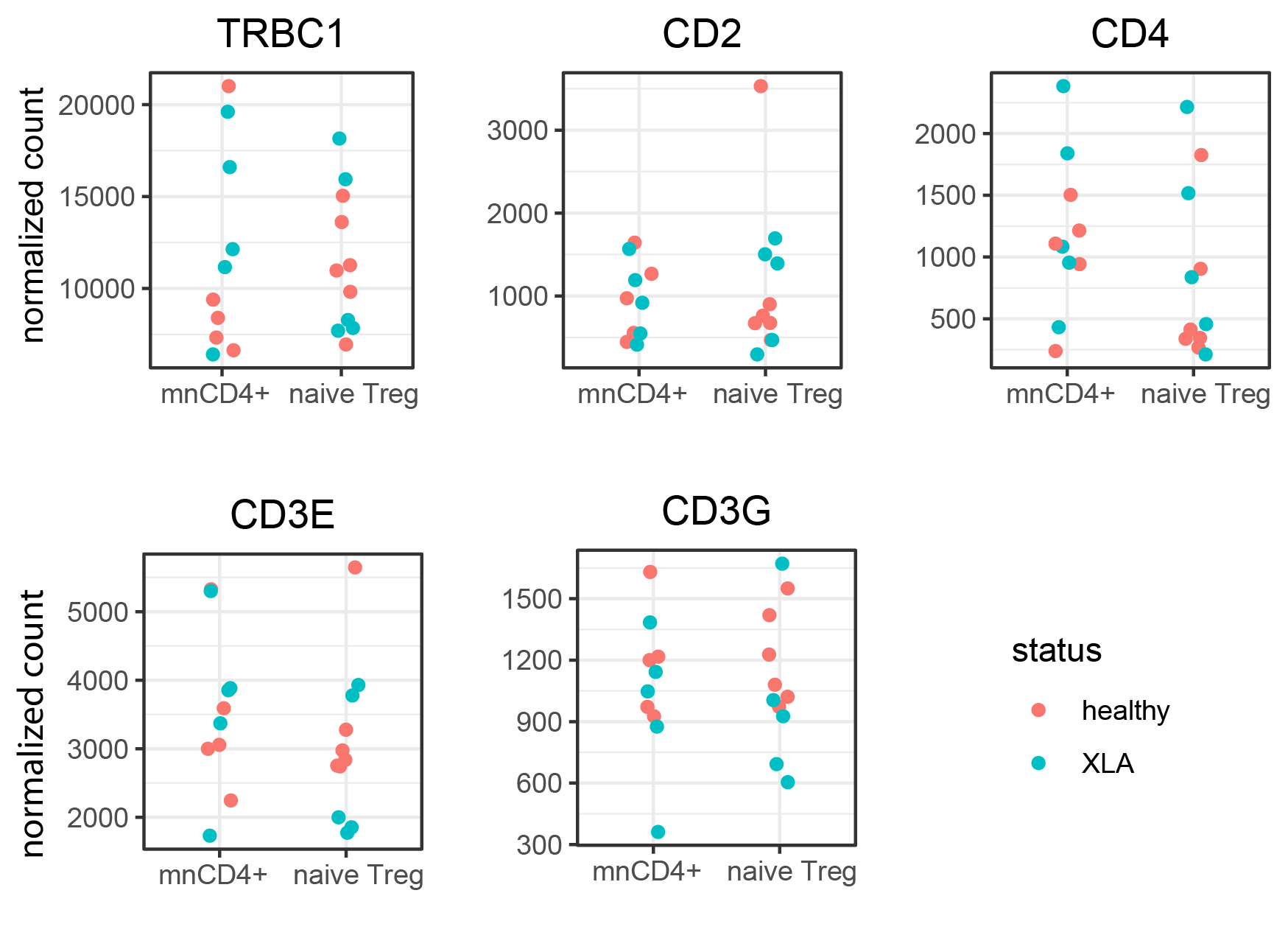


**Supplementary Figure S4. Expression of T-cell-specific genes in XLA and healthy donors.** Counts are normalized by DESeq2 (median of ratios method).


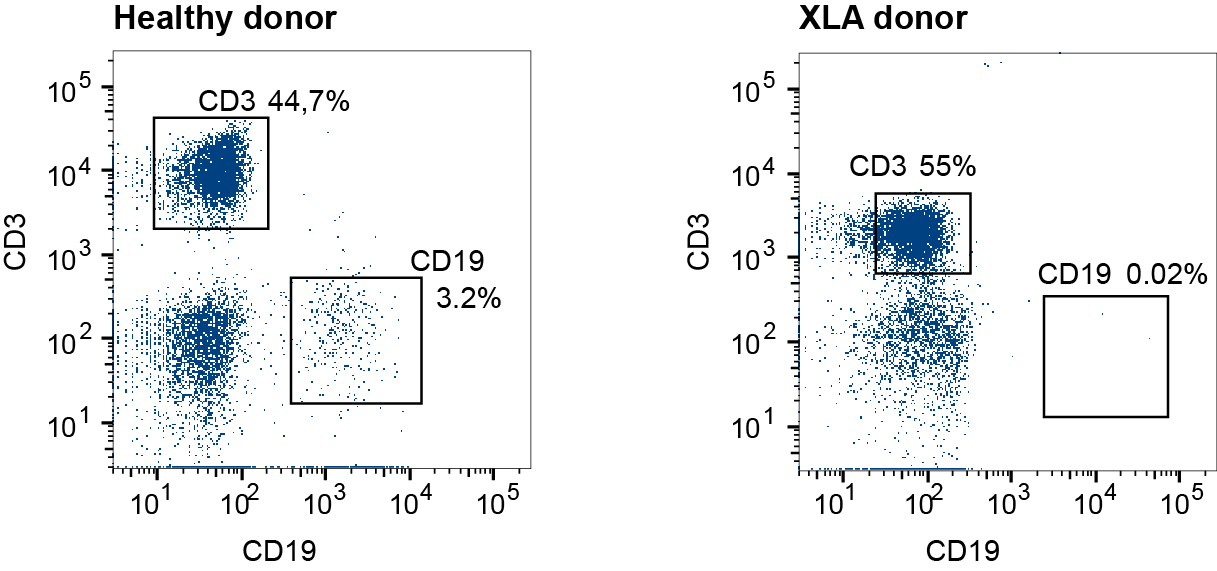


**Supplementary Figure S5. Representative staining for B-cell marker CD19.** PBMCs from young healthy (left) and XLA (right) donors were stained with antibodies against CD3 and CD19 to validate the XLA phenotype in patients. B cells were defined as CD3^-^CD19^+^.

**Supplementary Materials and Methods**

**Genotyping of XLA donors.**

High-throughput sequencing analysis was performed on 10 XLA patients. The pathogenic mutations were found, and two donors had additional benign mutations (X1, X3). No mutations in the BTK gene were detected in two donors (X2, X10) with apparent XLA symptoms, which probably reflects autosomal recessive agammaglobulinemia. In contrast, patient X8, with a diagnosis of PID, had a pathogenic mutation in BTK that confirmed the XLA diagnosis. Two patients had the same deletion (Cys145>AlafsTer31/c.433delA). Each of four benign mutations (c.1889G>A, c.1350-29T>C, c.71T>C/Glu24>Gly, and c.22T>C) were detected in more than two patients. Three novel mutations were identified: two deletions (NP_000052.1:p.Met570del/NM_000061.2:c.1708_1710delCAT and p.Cys145Alafs*31/NM_000061.2:c.433delA) and one SNP (NM_000061.2:c.1909-1C>T). These novel variants were predicted to alter highly-conserved amino acids and were submitted to the Leiden Open Variation Database (LOVD).

**Cell sorting**

For memory and naive CD4^+^ and CD8^+^ (defined as CD3^+^ CD4^-^) T cell sorting, PBMCs were isolated by standard Ficoll density gradient centrifugation, washed twice, and stained with fluorescently-labeled antibodies CD3-APC-Cy7 (clone UCHT1), CD4-PE (clone RPA-T4), CD45RA-FITC (clone JS-83), and CD27-eFluor450 (clone O323) in the dark. Forward and side scatter were used to exclude dead cells, cell aggregates, and cell debris. For RTE, naive T_reg_, and mnCD4^+^ T cell sorting, CD4^+^ T cells were purified on a Ficoll density gradient with RosetteSep Human CD4 enrichment cocktail (STEMCELL) and stained with CD4-APC-Cy7 (clone 13B8.2), CD25-PE (clone BC96), CD31-AlexaFluor647 (clone WM59), CD27-eFluor450 (clone O323), and CD45RA-FITC (clone JS-83) antibodies. From the naïve T cells parent gate, the following helper T-cells were selected: naive T_reg_ (CD4^+^CD45RA^+^CD27^+^CD25^+^) and mnCD4^+^ (CD4^+^CD45RA^+^CD27^+^CD25^-^CD31^-^), RTE (CD4^+^CD45RA^+^CD27^+^CD25^-^CD31^+^). To confirm the XLA phenotype of patients, we performed a flow cytometry analysis of PBMCs stained with CD3-APC-Cy7 (clone UCHT1) and CD19-FITC (clone HIB19). Flow cytometry and cell sorting was performed on BD FACSAria III, data was analyzed using BD FACSDiva software.

**Sequencing and data treatment.**

Raw sequencing data were analyzed using MIGEC software v.1.2.9 [[3]](https://paperpile.com/c/9bmjBK/MQFL). to group sequencing reads carrying the same UMI and thus covering the same cDNA molecule (SI Table 2, 3). Briefly, the Checkout utility was used for data demultiplexing and UMI sequence extraction, yielding ~90% sample barcode matching, and the data were then assembled using the Assemble utility with erroneous UMI filtering option. Over-sequencing threshold - minimal required number of reads per UMI - was set as 1, for all tasks except for the analysis of plasticity and publicity metrics, where it was set as 2. The latter types of analysis are sensitive to even minor cross-sample contaminants that may happen on the solid phase of Illumina. However, such contaminations are efficiently removed using reads-per-UMI threshold. In-frame TCR alpha and beta CDR3 repertoires were extracted using MiXCR version 2.1.1 software. Normalisation, data transformation, in-depth analysis, statistics calculations and visualization were performed using VDJtools version 1.2.1 software and R scripts, using libraries ggplot2, ggpubr and ggbiplot.

The input of each clonotype was proportional to its frequency within a sample. Basic analysis included 6 of the characteristics provided in VDJtools software, which were previously selected in various TCR and IG repertoire datasets [[1,2](https://paperpile.com/c/9bmjBK/DFYa)]: CDR3 length, number of added random (N) nucleotides, interaction "strength", Kidera factor 4 (hydrophobicity), "volume", "surface".

**Kidera** factor 4 reflects the abundance of hydrophobic amino acids with reversed scale, lower values refer to more hydrophobic amino acids. The “surface” characteristic in VDJtools reflects the relative abundance of amino acids which do not change their accessibility when TCR is in contact with pMHC in the whole CDR3 region or in the selected region of interest.

To calculate the mean **charge** of CDR3β, each amino acid residue was assigned a number depending on the charge: (+1) for R, H, K; (-1) for D, E; and (0) for the remaining residues. The sum was then divided by the number of amino acid residues. To calculate the mean **strength** of CDR3β, each amino acid residue was assigned a value depending on whether it is strongly binding (1) or not (0). The set of strongly binding amino acid residues includes F, L, I, M, V, W, Y, which are involved in hydrophobic, Van der Waals, and other interactions [5]. The presence of strongly-binding amino acids in CDR3 often leads to the removal of T cells during negative selection [6].

**Chao1** was calculated to estimate changes in repertoire diversity. This statistic is calculated using the formula $сhao1=S_{obs}+\frac{{F_{1}}^{2}}{2F_{2}}$ , where S_obs is the number of clones in the sample, F_1 is the number of clones met only once, and F_2 is the number of clones met twice. Chao1 estimates overall diversity, considering unmet clones. Therefore, a term is added to the total number of clones in the sample that estimates the number of unmet clones. It is assumed that the more clones that are met once in relation to clones met twice, the more clones that are not reflected in the sample. To estimate the presence of clonal expansion we used the **normalized Shannon-Weiner index**  $D=\frac{-\sum_{i}^{S} p_{ilogp_{i}}}{logS}$, where $S$ is the number of clones, and $p$ is the frequency of clones. As one can see from the formula, the index reflects normalized information entropy. The closer the index is to one, the more homogeneous the sample. If large clonotypes are present in the sample, then the index deviates significantly from one. The average volume was calculated with values for each amino acid residue shown in **Supplementary Table S5** [7].

**Supplementary Table S5. Values for calculating volume parameter by VDJtools.**

| Amino acid | A | C | D | E | F | G | H | I | K | L |
| --- | --- | --- | --- | --- | --- | --- | --- | --- | --- | --- |
| Volume | 67 | 86 | 91 | 109 | 135 | 48 | 118 | 124 | 135 | 124 |
| Amino acid | M | N | P | Q | R | S | T | V | W | Y |
| Volume | 124 | 96 | 90 | 114 | 148 | 73 | 93 | 105 | 163 | 141 |

Following data on the most abundant V segments were excluded: TRBV28 and TRBV29-1 naive T_reg_ for a single XLA patient from analysis of CDR3β length; TRBV28 and TRBV29-1 naive T_reg_ for a single XLA patient from analysis of added nucleotides in CDR3β; TRBV28 and TRBV29-1 naive Treg are excluded for a single XLA patient; TRBV6-5 naive T_reg_ is excluded for a single XLA patient from analysis of estimated average number of strongly binding amino acids.

**RNA-seq library preparation and analysis.**

The quality control of raw sequencing data and pre-treatment was performed with FastQC. RNA-Seq raw reads were aligned to the Ensembl reference genome (Homo_sapience.GRCh38.dna.primary_assembly.fa) with genome indexes generated using the Ensembl annotated transcripts (Homo_sapience.GRCh38.99.gtf) with STAR (version 2.7.5a). Read counts were generated with --quantMode GeneCounts option. A read is counted if it overlaps only one gene. Both ends of the paired-end read are checked for overlaps. Read-counts were further analyzed by DESeq2 (version 1.24.0) for differential expression according to the manual recommendations. The batch effect of different runs was removed by Limma. We measured the effect of the condition or cell type, controlling the batch differences. FDR cutoff is 0.05. The ‘ashr’ shrinkage estimator was used for ranking and visualization. False discovery rates: a new deal. Biostatistics, 18:2. [[4]](https://paperpile.com/c/9bmjBK/WwQ4). Raw data is available at by PRJNA752868 in the SRA (NCBI).

**Supplementary References**

1. [Davydov AN, Obraztsova AS, Lebedin MY, Turchaninova MA, Staroverov DB, Merzlyak EM, Sharonov GV, Kladova O, Shugay M, Britanova OV, et al. 2018. Comparative Analysis of B-Cell Receptor Repertoires Induced by Live Yellow Fever Vaccine in Young and Middle-Age Donors. *Front Immunol* **9**: 2309.](http://paperpile.com/b/9bmjBK/Qkdc)
2. [Izraelson M, Nakonechnaya TO, Moltedo B, Egorov ES, Kasatskaya SA, Putintseva EV, Mamedov IZ, Staroverov DB, Shemiakina II, Zakharova MY, et al. 2018. Comparative analysis of murine T-cell receptor repertoires. *Immunology* **153**: 133–144.](http://paperpile.com/b/9bmjBK/DFYa)
3. [Shugay M, Britanova OV, Merzlyak EM, Turchaninova MA, Mamedov IZ, Tuganbaev TR, Bolotin DA, Staroverov DB, Putintseva EV, Plevova K, et al. 2014. Towards error-free profiling of immune repertoires. *Nat Methods* **11**: 653–655.](http://paperpile.com/b/9bmjBK/MQFL)
4. [Stephens M. 2017. False discovery rates: a new deal. *Biostatistics* **18**: 275–294](http://paperpile.com/b/9bmjBK/WwQ4)
5. [Chakrabarti P, Bhattacharyya R. 2007. Geometry of nonbonded interactions involving planar groups in proteins. *Prog Biophys Mol Biol.* **95**:83–137.](http://paperpile.com/b/SsXixT/clSe)
6. [Kosmrlj A, Jha AK, Huseby ES, Kardar M, Chakraborty AK. 2008. How the thymus designs antigen-specific and self-tolerant T cell receptor sequences. *Proc Natl Acad Sci U S A*. **105**:16671–6.](http://paperpile.com/b/SsXixT/JIWx)
7. Pommié C, Levadoux S, Sabatier R, Lefranc G, Lefranc MP. 2004. IMGT standardized criteria for statistical analysis of immunoglobulin V-REGION amino acid properties. *J Mol Recognit.* **17**:17–32.
